# Enhance the Concrete Crack Classification Based on a Novel Multi-Stage YOLOV10-ViT Framework

**DOI:** 10.3390/s24248095

**Published:** 2024-12-19

**Authors:** Ali Mahmoud Mayya, Nizar Faisal Alkayem

**Affiliations:** 1 College of Automation and College of Artificial Intelligence, Nanjing University of Posts and Telecommunications, Nanjing 210046, China; 2 Computer and Automatic Control Engineering Department, Faculty of Mechanical and Electrical Engineering, Tishreen University, Lattakia 2230, Syria; ali.mayya@tishreen.edu.sy

**Keywords:** crack detection, crack classification, deep learning, YOLOV10, vision transformer, multi-stage model

## Abstract

Early identification of concrete cracks and multi-class detection can help to avoid future deformation or collapse in concrete structures. Available traditional detection and methodologies require enormous effort and time. To overcome such difficulties, current vision-based deep learning models can effectively detect and classify various concrete cracks. This study introduces a novel multi-stage deep learning framework for crack detection and type classification. First, the recently developed YOLOV10 model is trained to detect possible defective regions in concrete images. After that, a modified vision transformer (ViT) model is trained to classify concrete images into three main types: normal, simple cracks, and multi-branched cracks. The evaluation process includes feeding concrete test images into the trained YOLOV10 model, identifying the possible defect regions, and finally delivering the detected regions into the trained ViT model, which decides the appropriate crack type of those detected regions. Experiments are conducted using the individual ViT model and the proposed multi-stage framework. To improve the generation ability, multi-source datasets of concrete structures are used. For the classification part, a concrete crack dataset consisting of 12,000 images of three classes is utilized, while for the detection part, a dataset composed of various materials from historical buildings containing 1116 concrete images with their corresponding bounding boxes, is utilized. Results prove that the proposed multi-stage model accurately classifies crack types with 90.67% precision, 90.03% recall, and 90.34% F1-score. The results also show that the proposed model outperforms the individual classification model by 10.9%, 19.99%, and 19.2% for precision, recall, and F1-score, respectively. The proposed multi-stage YOLOV10-ViT model can be integrated into the construction systems which are based on crack materials to obtain early warning of possible future deformation in concrete structures.

## 1. Introduction

Concrete is a construction material that has an essential role in building due to its characteristics like low cost, durability, strength, versatility, etc. [1,2,3,4]. However, due to many service and environmental factors, concrete may be exposed to different types of damage such as cracks, defects, deformation, spalling, and scaling [5,6,7]. These issues affect the reliability of structures and may even result in structural collapse especially when minor damage occurrence is ignored for a long time [8]. Therefore, early detection and classification of concrete crack types can limit these problems and maintain the concrete structures [2,9,10,11,12,13]. Moreover, concrete crack detection helps to ensure building safety by the early detection and warning of the potential concrete failure that can happen due to the frequent cracks in concrete material. Early detection and type classification of such concrete cracks raise warnings and provide possible treatment according to crack type leading to more structure safety [14]. Manual methodologies to detect and classify cracks are time-consuming and require too much effort. However, current artificial intelligence (AI) technologies, including machine learning (ML) and deep learning (DL), can facilitate the concrete crack detection and classification task as well as further save human lives [3,15].

Current state-of-the-art methodologies utilize many DL architectures due to their efficiency and ability to learn from large amounts of concrete images [4,16]. Most of these studies have considered only one task (detection, segmentation, or classification). Too few researchers took into consideration building multi-stage concrete crack detection models.

### 1.1. Related Work

Several recent articles studied the utilization of convolutional neural; networks CNNs for concrete crack identification with the use of the individual YOLO or Vision transformers (ViT) models for either region-based detection or image classification [17,18]. Among the recent studies, a multi-scale crack binary classification method was proposed by Yadav et al. [19]. Their methodology depended on three feature extraction phases; the local binary pattern, the simple linear iterative clustering, and the 3ScaleNetwork. The experiments were applied to the “Historical_Building_Crack_2019” consisting of 3886 images (757 of them are of the crack class). Their methodology achieves 98.9%, 99.18%, and 99.69% for precision, recall, and accuracy, respectively. However, the training and validation accuracy curves of their study showed relativity unstable performance. In another study by Chen et al. [20], a multi-resolution semantic segmentation with convolutional neural networks was proposed. They applied the feature aggregation method to enhance the ability of CNN models to detect cracks. A dataset of 2000 images of bridge, dam, and spillway materials was utilized. The results showed 94.51% and 86.39% as precision and recall. ViTs were utilized by many studies in the field of concrete crack classification due to their high performance and lightweight architecture. Yadav et al. [21] fused the CNNs with the ViT model to improve crack detection in building concrete. The Historical Building Crack2019, SDTNET2018, and DS3 concrete datasets were utilized and the results showed a precision of 98.6%, 98.93%, and 99.33% using those three datasets, respectively. Another study by Shahin et al. [22] proposed a hybrid ViT model to improve concrete crack detection. They achieved an accuracy of 99% on the “Concrete Crack Images for Classification” containing 20,000 images of crack and 20,000 images of no-crack types. Residual deep learning was also considered in some pieces of research due to its efficiency. Wang et al. [23] suggested using the ResNet18 model to identify crack and non-crack images using 2000 images of four types (non-crack, transverse crack, vertical crack, and Craquelure). The trained ResNet18 model achieved an accuracy of only 90% using the test set. Ensemble learning of many DL models and transfer-learning ensemble-based methodology was also proposed in [24] for the aim of bridge crack detection. They made many ensembles with different methodologies and achieved precision, recall, and accuracy ratios of 97.64%, 99.41%, and 98.62%, respectively on the “SDNET2018” dataset which contains 56,092 images of different bridge, wall, and pavement materials. Pavement crack detection was addressed in a study by Wang et al. [25] using a hybrid DL model of a weakly-supervised transformer model with an attention mechanism. They achieved a precision value of 95.1%, a recall value of 95.9%, and a mIoU value of 91.3% on the DeepCrack dataset which consists of 537 high-resolution images. Xception and Vanilla models were proposed by Abubakr et al. [26] to classify concrete images into non-crack, crack, corrosion, efflorescence, spalling, exposed bars, and spallation types. They utilized the concrete defect bridge image (CODEBRIM) dataset consisting of 10,810 images. They achieved an accuracy of 94.95% of the Xception model. To learn crack features at different scales, Russel and Selvaraj [27] proposed a multi-scale CNN-based architecture to enhance crack detection accuracy. They evaluated their methodology using the “Middle East Technical University” dataset consisting of 20,000 crack and non-crack images. The outcomes showed high performance of 99.3%, 99.9%, and 99.96% for precision, recall, and accuracy, respectively.

You only look once (YOLO) model and its multiple versions were significantly utilized for the aim of crack detection. An improved YOLOV8 model was utilized by Dong et al. [28]. They added the separable kernel attention mechanism to improve the detection process. The RDD2022 and Wall Crack datasets were utilized and the results registered an increase in average precision by 15.2% and 12.3% on both datasets. Wang et al. [29] integrated the ViT model as a backbone of the YOLOV8 model for the aim of asphalt pavement crack detection. They achieved an average precision of 0.872 on a small dataset of 1994 asphalt pavement images. The utilization of ViT inside the YOLOV8 model improved the performance of crack detection. Multi-stage system of road crack detection and segmentation based on CNN was proposed by Nguyen et al. [30]. In the detection part, they utilized a CNN-based simple model, while in the segmentation, they implemented a U-Net model. They achieved an F1-score of 0.91 on the DeepCrack dataset consisting of 537 images, and the CrackIT dataset containing 84 pavement surface images. They created a ground truth for this dataset. Sohaib et al. [31] developed a similar detection and segmentation framework based on YOLOV8 and bounding box refinement methodology. A dataset of 6315 concrete images was utilized and the study achieved 92.33%, 90.02%, and 89.5% as precision, recall, and mean average precision (mAP), respectively. They also evaluated their approach on the Pavement Crack dataset and achieved 89.62%, 87.91%, and 88.76% for precision, recall, and F1-score, respectively. Dai et al. [32] presented a multi-stage damage detection methodology based on machine learning and piezoelectric singular feature analysis. They also trained a modified back propagation-based network on the extracted feature vectors. A signal-based dataset of 1344 signals was utilized. The study achieved a classification accuracy of 92%, a precision of 95.4%, and a recall of 92.9%. Multi-task crack segmentation and quantification DL model was introduced by Chen et al. [33]. The proposed multi-task U-Net and multi-task DeepLabV3+ models improved the concrete crack quantification by above 2% using the DeepCrack dataset. A three-stage DL framework was designed by Huang et al. [34] to efficiently detect the crack width of concrete cracks. The three stages were: image segmentation, fractal dimension to remove false responses, and the crack-width calculation. They compared many segmentation architectures (U-Net, ResU-Net, Seg-Net, etc.) and found that ResU-Net provided the best performance.

From the above literature survey, some observations can be concluded as:

- The recent literature lacks the effective implementation of the idea of multi-stage detection and multi-class classification based on hybrid lightweight models.
- Current and historical studies often focus on specific tasks (either detection or classification).
- The modern ViT classification models outperform the traditional CNNs and available transfer-learning models and guarantee robust and efficient performance.
- The most recent YOLO model (YOLOV10) model has not yet received high attention in the field of crack detection and still needs more investigations.
- All previous attempts to develop multi-stage systems focus on one mission (detection, classification, segmentation), and few studies considered the detection-classification issue. However, the literature is short of region-based detection and classification frameworks.

### 1.2. Contribution

This study aims to develop a novel deep learning-based framework for the detection and multi-class classification of concrete cracks. The study introduces a multi-stage model based on the detection of the state-of-the-art YOLOV10 model and the efficient classification capability of the ViT model into one combined hybrid detection and classification model. The main contribution of the current study can be concluded as:

- Creating a novel deep learning framework utilizing the capability of two detection and classification models (YOLOV10 and ViT) called the “YOLOV10-ViT” framework.
- Utilizing of new concrete crack detection dataset for training the YOLOV10 model, and depending on a multi-class classification concrete crack classification dataset for training the ViT model. The aim of the combination of two-source datasets to train and test the proposed YOLOV10-ViT model provide more efficient identification with boosted generalization abilities.
- Comparison of the performance with and without the pre-detection stage of the YOLOV10 model to show the superiority of the proposed multi-stage technique.

The rest of the paper is organized as follows: First, the materials (datasets) and the proposed methodology are presented and illustrated in Section 2. Then, the main results of both individual and multi-stage models are introduced in Section 3. Later, Section 4 discusses the outcomes and the various performance comparisons to judge the robustness and efficiency of the proposed methodology. Finally, the main conclusion and future perspectives are drawn in Section 5.

## 2. Materials and Methods

### 2.1. Main Methodology

**Figure 1:**
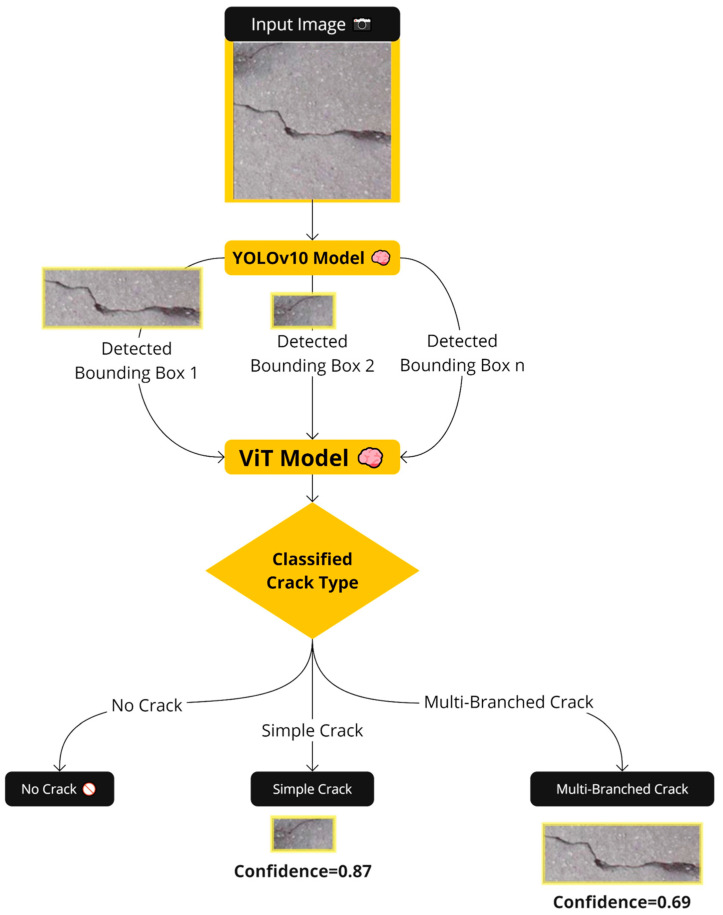
Flowchart of the proposed multi-stage crack detection and classification framework.

This study involves three main stages within a novel multi-stage detection classification framework. First, a vision transformer model (ViT) is trained using a concrete crack multi-class classification dataset to configure the classification thread. Second, a “You Only Look Once” YOLOV10 model is trained based on a crack-detection dataset resulting in the detection part. The third branch combines the trained YOLOV10 model and the trained ViT model so that the detected regions of the YOLOV10 model are fed into the ViT model to individually classify them as illustrated in Figure 1.

### 2.2. The Datasets

**Figure 2:**
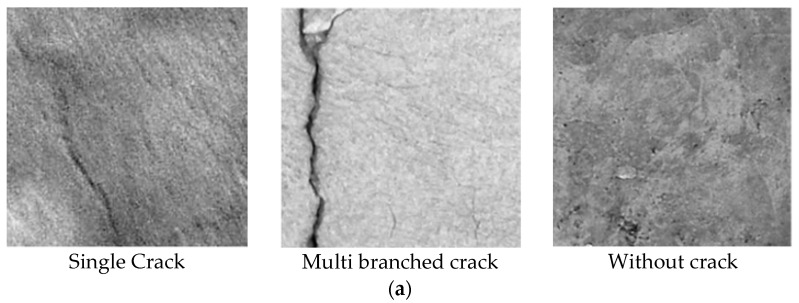

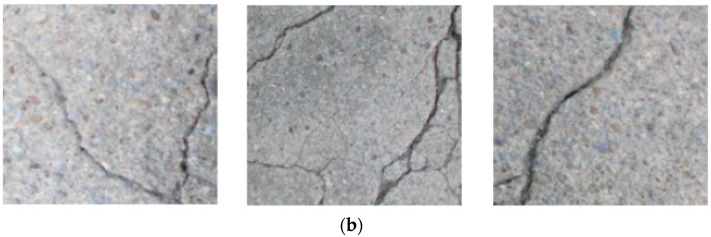
Some samples of the utilized: (**a**) concrete classification and (**b**) detection datasets.

In this section, the datasets utilized in the three sections of this study will be introduced. In the classification branch, the “Cracks In Concrete Structures (CICS)” [35] concrete multi-class classification-based dataset is utilized. This dataset contains 12,000 images distributed equally on three classes: ‘single crack’, ‘multi-branched crack’, and ‘without crack’. The images have a resolution of 330 × 330 and were collected from various concrete structures (concrete cement type 2). For the second detection part of the proposed methodology, another concrete crack detection dataset “Crack in various Materials from Historic Buildings” [36,37] is used. This dataset is one of the newest crack detection datasets consisting of different sub-sets. In our study, the sub-set “concrete crack detection” consisting of 1116 concrete images will be utilized. The dataset is originally split into a training set (70%), a validation set (20%), and a test set (10%). Figure 2 includes samples of the utilized classification and detection concrete datasets.

### 2.3. Deep Learning Methodologies

**Figure 3:**
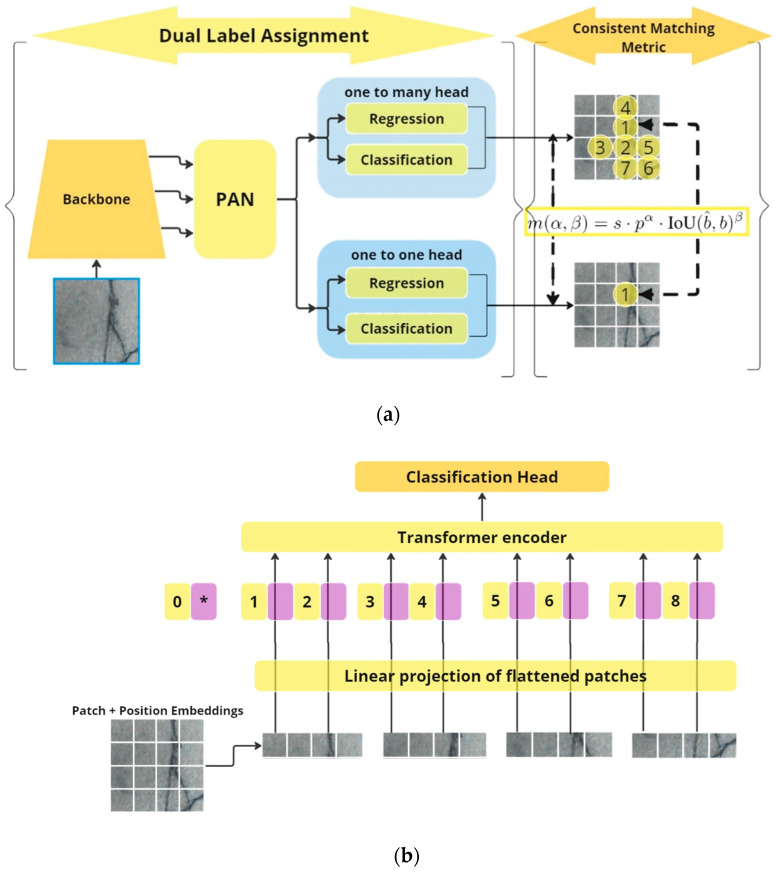

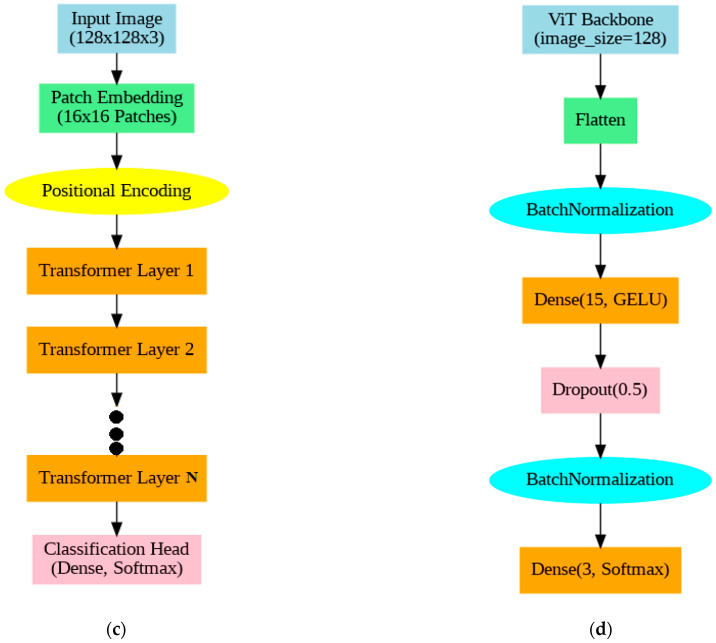
Deep learning models: (**a**) YOLOV10 detection model; (**b**,**c**) ViT backbone (feature extraction head); and (**d**) ViT backbone with a classification part; * is the extra learnable [class] embedding.

The main two DL architectures utilized in this study are the YOLOV10 and the ViT models. The architecture of the YOLOV10 model is shown in Figure 3a, while the main architecture of the ViT feature extraction part (encoder-based architecture) is illustrated in Figure 3b, and the entire ViT backbone with the proposed classification head is shown in Figure 3c.

#### 2.3.1. YOLOV10 Detection Model

The YOLOV10 model follows the original architecture of the YOLO model with remarkable enhancement [38,39,40]. In the ordinary YOLO models, the one-to-many methodology was mainly utilized leading to many possible bounding boxes (and even many false positives). Those models used the non-maximum supersession (NMS) to eliminate redundant bounding boxes [38,39]. However, false positives are still noticed. In the YOLOV10 model, both one-to-many and one-to-one heads are proposed to overcome this problem. In a one-to-one strategy, a single prediction (one bounding box) is assigned to each ground truth, which eliminates the duplicate predictions and the need for the NMS stage [41]. Through the training process, both one-to-one and one-to-many heads are optimized for better prediction. Moreover, in the prediction (evaluation), the YOLOV10 model utilizes the one-to-one head only eliminating the need for NMS and giving more robust predictions [42,43]. This strategy is called the dual label assignment with NMS-free training which is shown in the first left part of Figure 3a. In the right part of Figure 3a, the consistent matching metric is utilized to check the intersection between the predicted bounding boxes and the actual ground truth, which is mathematically computed using the intersection of union (IoU) formula shown in Equation (1) [39].

(1) $$m\left(\alpha ,\beta \right)=s\cdot p^{\alpha }\cdot IoU{\left(\hat{b},b\right)}^{\beta },$$

where $\hat{b},b$ are the original and predicted bounding boxes, and α, β are the hyperparameters that must be learned to balance the effect of the semantic prediction and the location regression tasks. While ‘s’ refers to the spatial prior that indicates if the anchor point of the predicted bounding boxes is inside the instance, ‘p’ denotes the classification score [39]. The main architecture of the head part of the YOLOV10 model follows the same architecture of the YOLO model with some lightweight calculations. The “Spatial-Channel Decoupled Downsampling” (SCDown) block is the down-sampling block that increases the number of channels and reduces the number of spatial dimensions. In the YOLOV10 model, this downsampling operation is decoupled allowing less computations and more information. Besides that, the YOLOV10 model presented a new block called a compact inverted block (CIB) with better cost-effectiveness by using depthwise convolutions [39,43]. Moreover, the Partial selection attention (PSA) block is responsible for dividing channels with a 1x1 convolution, then applying the self-attention to specific parts of the features, and fusion of multiple feature maps. The PSA block also applies the dimensionality reduction which minimizes the computational complexity (especially since the PSA is only placed after the fourth stage of the YOLOV10 model).

#### 2.3.2. Vision Transformer Model

ViT is a type of transformer model that accepts its input as an image and produces its output as a classification [44,45]. Figure 3b,c shows the architecture of the ViT backbone [46]. The input image I_x,y_ is divided into patches with size *S* × *S* which will be further flattened. The flattened patches are then passed to a linear embedding layer where they will be treated as sequences (tokens) [47,48]. Then, positional embedding information is added to the embedding to provide the model with spatial information. The next step is to feed these positional embedding patches into a transformer encoder model [49]. The encoder consists of three main parts: the multi-head attention layers (transformer encoder) which is responsible for extracting image features with attention mechanism (giving attention to specific parts of the input) leading to a better learning operation, the add&norm module which adds residual connection to the model to avoid vanishing gradients, besides the normalization to improve the training stability, and the multi-layer perceptron (MLP) which is used as the classification head in the transformer model [50]. However, in this study, the MLP part is dropped and replaced by a specific classification head shown in Figure 3d. The classification head consists of flattening, batch normalization, a dense layer with 15 neurons, dropout with 50% percentage, another batch normalization, and a dense classification layer with three neurons (three classes). The input images and their corresponding labels will be fed into the model in a supervised learning way.

#### 2.3.3. Training Options

YOLOV10 model includes an essential step which is the data albumentation (augmentation) step, in which some transformations are applied to images to both increase the training size and make the training process robust against changes. For the YOLOV10 model, the concrete crack dataset is first pre-processed by the model itself via applying data albumentation (augmentation) operations on the training set as the following: Blur (probability = 0.01, blur limit = (3,7)), median Blur(probability = 0.01, blur limit = (3,7)), contrast limited adaptive histogram equalization CLAHE(*p* = 0.01, clip limit = (1, 4.0), and tile grid size = (8, 8)).

**Figure 4:**
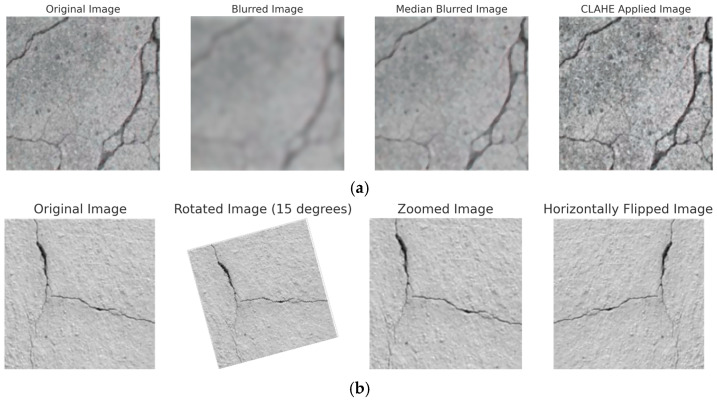
Samples of the training set of YOLOV10 and ViT model and their corresponding data (**a**) albumentation, (**b**) augmentation.

For the ViT model, the dataset is split into 80% as a training set and 20% as a test set. Then, the data augmentation operations are performed on the training set images, including random rotation in the range of 0^0^–15^0^, random zooming with a range of 0.15, and horizontal and vertical flips. Figure 4 includes examples of data augmentation and data albumentation of both ViT and YOLOV10 models on two training samples.

After preprocessing, splitting, and data augmentation operations, the YOLOV10 model and the ViT model are trained using the training options and parameters illustrated in Table 1 and Table 2.

#### 2.3.4. Performance Evaluation

Precision, recall, F1-score, and accuracy are the performance metrics that are utilized to assess the trained ViT performance. All these metrics are based on four basic calculations: true positives (TP), true negatives (TN), false positives (FP), and false negatives (FN) [51,52]. According to our problem, and considering a class-type-A out of three classes, TP represents the number of crack-type-A samples that are correctly classified as crack-type-A, while the TN denotes the number of samples that are not of the crack-type-A and the model correctly rejected them as non-Crack-Type-A. FPs represent the samples that are incorrectly accepted in a specific class (crack-type-A), while FNs are the incorrectly rejected ones. According to previous calculations, precision, recall, F1-score, and accuracy are given as Equations (2)–(5) show [53]. Precision indicates how many of the positively classified samples are actually positive. A high precision value indicates a low false positive rate. Recall (sensitivity) indicates how many of the real positive samples were successfully identified by the model, so a high recall indicates a low false negative rate. Accuracy, on the other hand, takes into consideration all true positives and true negatives summation to the entire number of samples (TP, TN, FP, and FN) so it gives an overall performance of the model.

(2) $$Precision=\frac{TP}{TP+FP}$$

(3) $$Recall=\frac{TP}{TP+FN}$$

(4) $$F1\text{-}score=\frac{2\;*\;Precision\;*\;Recall}{Precision+Recall}$$

(5) $$Accuracy=\frac{TP+TN}{TP+TN+FP+FN}$$

Confusion matrix (CM), receiver operating characteristic (ROC) curve, and area under the ROC curve (AUC) [54] are also utilized to evaluate the ViT performance to see the TP, TN, FP, and FN values per individual classes (CM), and see the tradeoff between true positive rate and false positive rate (ROC curve).

For the YOLOV10 model, the precision, recall, and mean approximate precision (mAP) values are computed to evaluate the accuracy of the detected bounding boxes. ‘mAP’ denotes the mean value of the average precision (AP) through many object classes. AP refers to the area under the precision-recall curve. While ‘mAP50’ computes the mean average precision at an intersection of union (IoU) threshold of 0.5 (50%), the ‘mAP50-95’ calculates the mean of average precision at different (IoU) values varying from 50% to 90% (which is a harder case) [55,56].

## 3. Results

In this section, the experiments that are performed within the three parts of this study will be presented. First, the results of the ViT classification model will be introduced; then, the YOLOV10 detection model’s results will be presented; and later, the main outcomes of the proposed multi-stage model will also be listed.

### 3.1. ViT Classification Model’s Results

**Figure 5:**
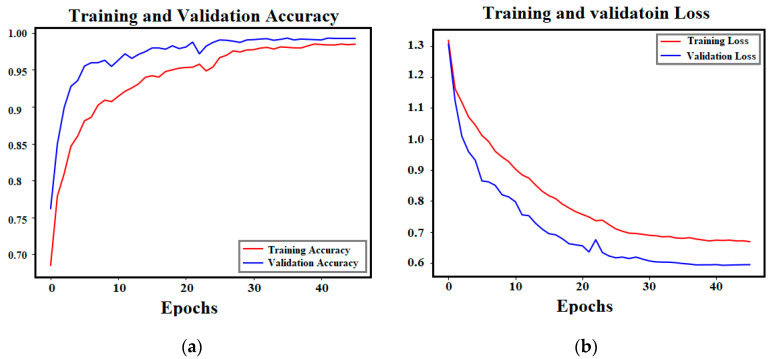
Training and validation accuracy and loss curves of the trained ViT model: (**a**) Accuracy; (**b**) Loss.

After training the ViT model on the multi-class network for 50 epochs, the validation accuracy of the trained model reached a score of 99.29%. The training and validation accuracy and loss curves of the trained ViT model are shown in Figure 5. The convergence between training and validation scores proves the robustness of the trained model without any possible overfitting or fake training.

The classification report of the trained ViT model is also presented in Table 3. High average scores of precision, recall, and F1-score indicate that the trained ViT model on the multi-crack dataset can correctly recognize the crack types and the non-crack (without crack) type. The ‘multi-branched crack’ class achieves the best precision score meaning that it has a smaller number of false positives compared to the other two classes. Similarly, the best-obtained recall value corresponds to the ‘multi-branched crack’ class, indicating that the ViT-trained model is better in identifying the correct ‘multi-branched crack’ class samples than the other two classes (this class has less number of false negative errors). However, the ‘simple crack’ and ‘without crack’ classes have similar precision and recall values to the ‘multi-branched’ class, and the weighted average and macro average scores are all high and similar. The F1-score of the trained ViT model reflects the convergence between precision and recall values and proves the training process’s robustness.

**Figure 6:**
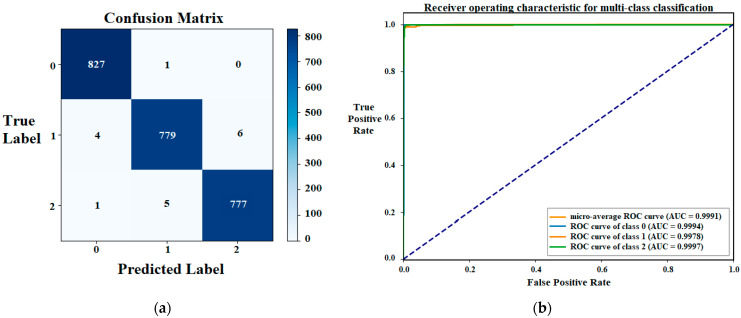
Confusion matrix and ROC plot for the trained ViT model using the multi-class crack dataset: (**a**) Confusion Matrix; (**b**) ROC plot.

The confusion matrix and the ROC plot are also derived for the three classes (Figure 6). The class ‘multi-branched crack’ has 1 false negative and 5 false positives, the class ‘single crack’ has 10 false negatives and 6 false positives, while the normal class ‘without crack’ contains 6 false negatives and 6 false positives. The AUC value for class 0 (multi-branched class) has a score of 0.9994 which is better than the ‘single class’ score. However, the ‘without crack’ class registers the best AUC value of 0.9997 and the average AUC value is 0.9991.

**Figure 7:**
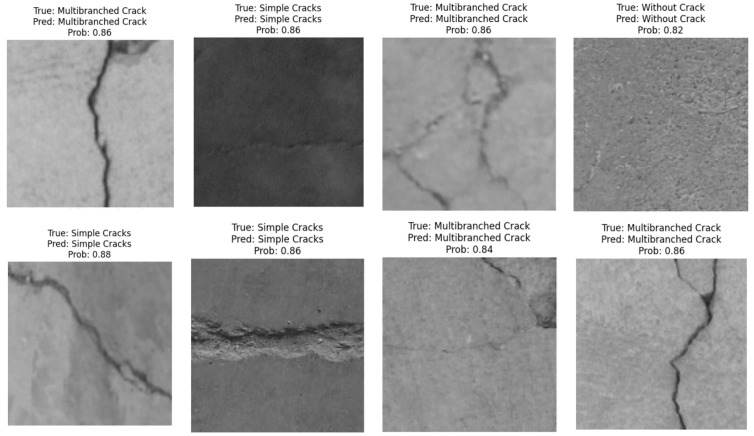
Correctly classified samples of the crack test set, their true labels, and the predicted labels with corresponding probabilities.

Figure 7 includes some successful visual experiments applied to various samples of the crack test set, taking into consideration all cases (without crack, single crack, and multi-branched crack).

### 3.2. YOLOV10 Detection Model’s Results

**Figure 8:**
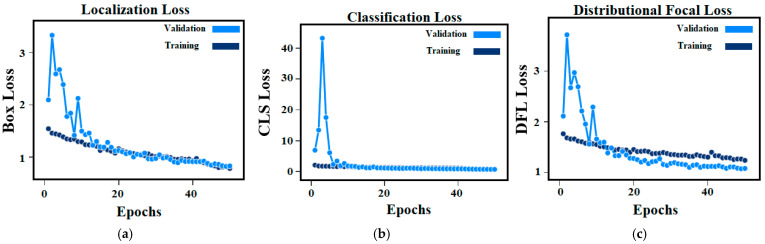
Trained YOLOV10 loss calculation per training epochs: (**a**) Box Loss; (**b**) CLS Loss; (**c**) DFL Loss.

The second part of the proposed methodology is the crack detection task which was performed based on the latest YOLO model (YOLOV10). The localization loss (Box Loss), the classification loss (CLS Loss), and the distributional focal loss (DFL Loss) of the trained YOLOV10 model during 50 training epochs are illustrated in Figure 8. In terms of box loss, Figure 8a shows that the YOLOV10 is learned well (the predicted bounding boxes and the ground truth ones are too close) since the box loss is decreased across epochs. Figure 8b indicates that the YOLOV10 model has learned to classify objects correctly with high confidence. The YOLOV0 model also handles the class imbalance effectively as shown in the DFL loss of Figure 8c. Moreover, the convergence between training and validation losses indicates no overfitting during the training process in all loss curves.

**Figure 9:**
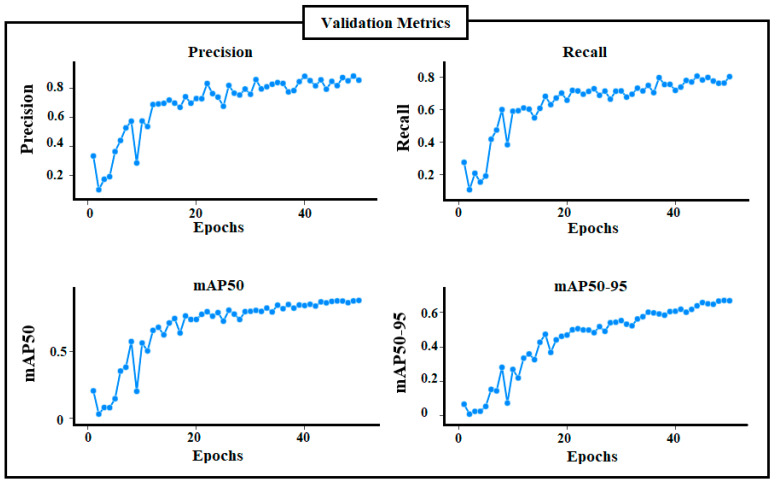
Validation metrics of the trained YOLOV10.

Figure 9 includes the validation metrics (precision, recall, mAP50, and mAP50-95) of the trained YOLOV10 model. All metrics are increased epoch by epoch indicating the ability of the trained model to correctly detect all crack regions (high recall), minimize the possibility of false positive non-crack regions (high precision), and balance between both detecting crack and rejecting non-crack correctly (high mAP). Metrics also indicate that the evaluation of the YOLOV10 model using the test set achieves 0.88296, 0.80405, 0.88139, and 0.66807 for precision, recall, mAP50 and mAP50-95, respectively.

**Figure 10:**
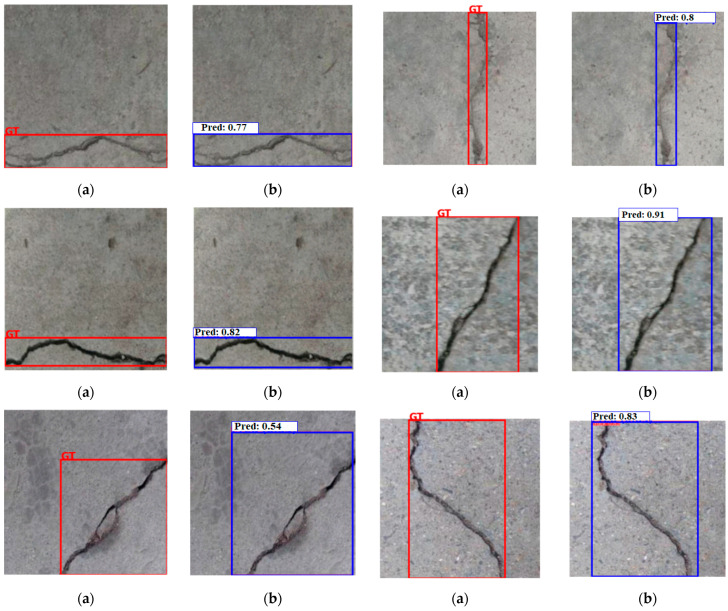
The predicted bounding boxes of the trained YOLOV10 model on some test examples: (**a**) Ground truth; (**b**) Prediction result.

Some test examples with their corresponding ground truth and the prediction results of the trained YOLOV10 model are listed in Figure 10. The trained YOLOV10 model correctly and accurately predicted the bounding boxes’ locations compared to the ground truth.

### 3.3. The Multi-Stage Model Results

**Figure 11:**
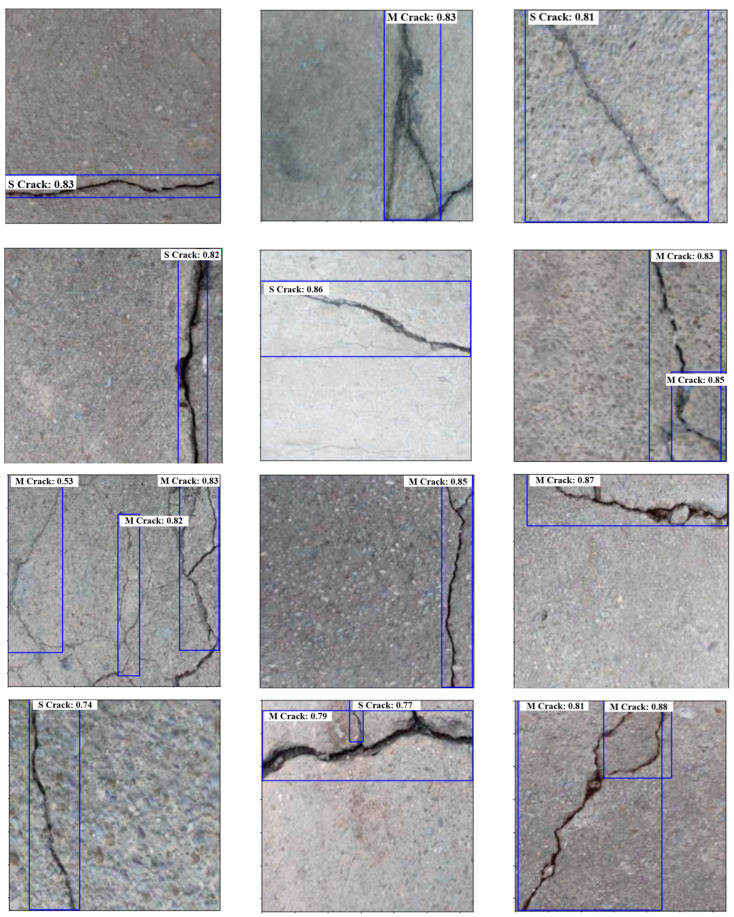
The outputs of the multi-stage crack prediction and classification model using some test samples.

This section presents the final outcomes of the proposed multi-stage model. The trained ViT and YOLOV10 models are both utilized in this step. First, the test image will be predicted by the YOLOV10 model, and then the extracted crack ROIs will be fed into the ViT model to classify them into two main parts; either simple crack or multi-branched crack. Figure 11 includes examples of the final prediction of the multi-stage model using some test samples. The prediction result contains information from both classification and detection models. From the ViT classification model, we get ‘S’ as the single crack class and ‘M’ as the multi-ranched crack, while from the YOLOV10 model, we obtain the bounding boxes, and the confidence level of the detection.

## 4. Discussion

The proposed YOLOV10-ViT model in this study has successfully enhanced the accuracy and reliability of concrete crack classification models. It has worked by integrating a pre-detection step utilizing the YOLOV10 model to initially detect all possible crack ROIs inside concrete images and then feed them into a trained ViT model to classify the crack types in the detected ROIs. The main goal has been to build a robust and accurate crack detection as well as an ROI-based classification tool that can assist building engineers in identifying and differentiating between crack types and normal concrete types, thereby improving concrete crack detection and classification accuracy and further building safety.

### 4.1. Individual Models Discussion

**Figure 12:**
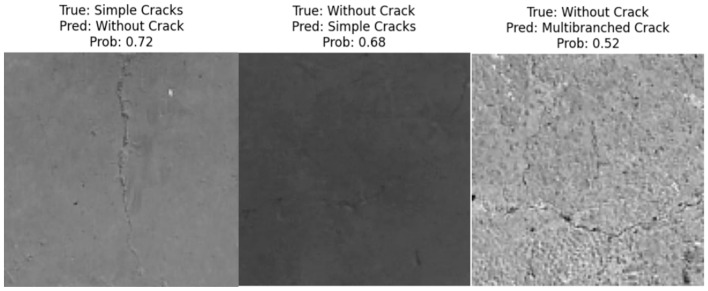
Misclassified samples of the crack test set.

The classification model (ViT) evaluation process has declared the stability and robustness of the trained model. The main challenge in the utilized dataset is the usage of three classes (single crack, multi-branch crack, and without crack). Despite the similarity in cases of single and multi-branched cracks in a lot of samples, the proposed ViT model achieved high precision, recall, and F1-score values for all classes. However, the distribution of errors across classes shows that only 0.12% of the ‘multi-branch’ crack type samples have been incorrectly classified as ‘single crack’ type. On the other hand, 0.5% of the ‘single’ crack type samples have been misclassified as ‘multi-branch’ crack type, and 0.76% of the ‘single’ crack type has been misclassified as ‘without crack’ type. None of the ‘multi-branched’ crack has been misclassified as ‘without class’ type. Considering the ‘without crack’ type, 0.12% of samples have been incorrectly classified as ‘multi-branched crack’, while 0.63% of them have been misclassified as ‘single crack’ type. Reviewing the dataset incorrectly classified samples, two main notable results have been achieved, the first one is that the percentage of the total misclassified samples has been only 0.71%. The second note is that some of the incorrectly ‘without crack’ classified samples contain tinny and limited-length crack or small defects regions, which has made the ViT model misclassify them as possible crack regions (see Figure 12).

The misclassification sample (First sample in Figure 12) contains a small crack but it is labeled in the original dataset as ‘Without crack’. The second sample in Figure 12 has a small defect leading the model to classify it as ‘single crack’. The last sample in Figure 12 contains a multi-branched crack but the crack is too thin so the model classifies it as ‘without crack’.

**Figure 13:**
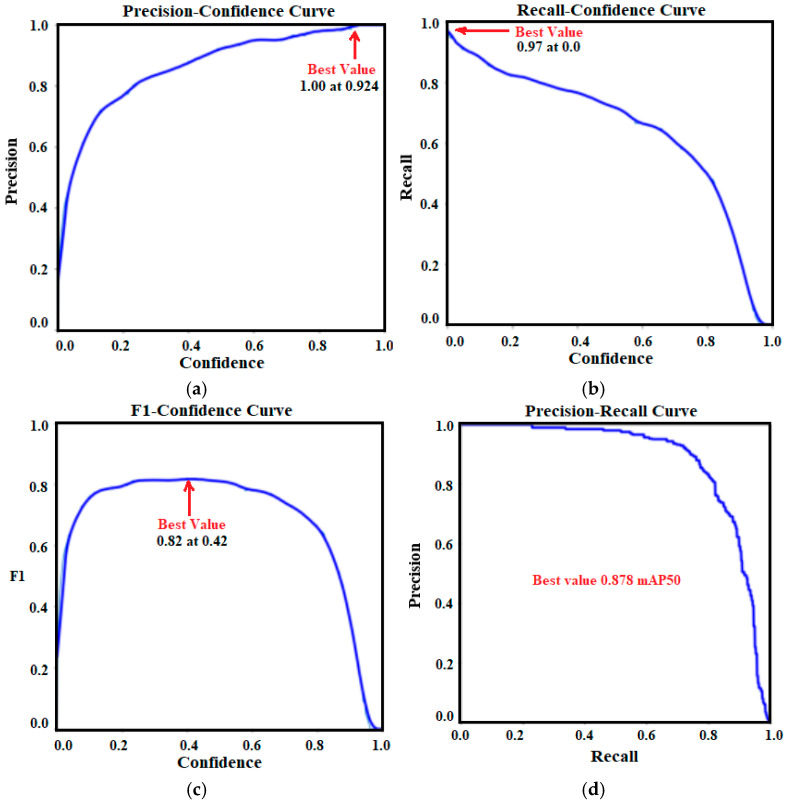
Evaluation metrics of the trained YOLOV10: (**a**) Precision-Confidence curve; (**b**) Recall-Confidence curve; (**c**) F1-Confidence curve; (**d**) Precision-Confidence curve.

The YOLOV10 model has been trained on a concrete crack detection dataset. Figure 13 includes the precision-recall tradeoff curves by which a detailed discussion of the trained YOLOV10 model can be inferred. Figure 13a illustrates the precision-confidence curve in which the precision is improved with more confidence level until reaching a value of one (100%) at a confidence threshold value of 0.924 (i.e., the trained YOLOV10 model makes its best positive predictions without any false positive errors when the confidence threshold is 0.924). Similarly, Figure 13b shows the recall-confidence curve in which the best recall value is 0.97 (97%) at a confidence level of 0 (i.e., the least false negative errors were registered at a confidence level of 0). Combining recall and precision together makes a better point of view (Figure 13c,d). The best confidence level by which the model has the least number of false positive and false negative errors is 0.42 where the F1-score value is 0.82 (82%). Moreover, the precision-recall curve indicates that the model maintains a good ability to correctly detect the true positives without showing a large number of false positives among a range of recall values. Figure 13d also indicates that the best mAP50 value is 0.878 (87.8%) indicating a good ability of the model to detect all possible crack ROIs. A small drop-off in precision at higher recall values (Figure 13d) indicates that as the trained YOLOV10 model tries to capture all possible positives, it starts to include some false positives. Overall, the trained YOLOV10 model using the concrete crack dataset can effectively balance precision and recall, which makes it trustfully suitable for the crack detection field.

### 4.2. Multi-Stage Model Results’ Discussion

**Figure 14:**
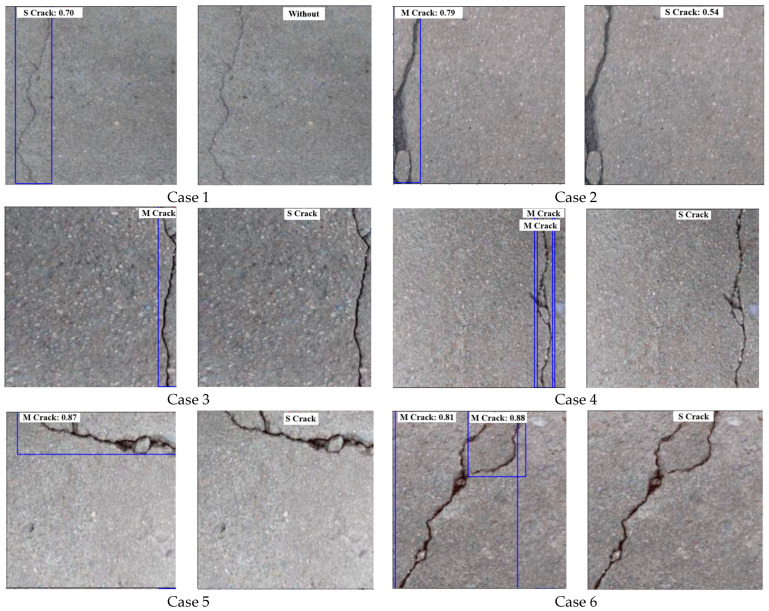

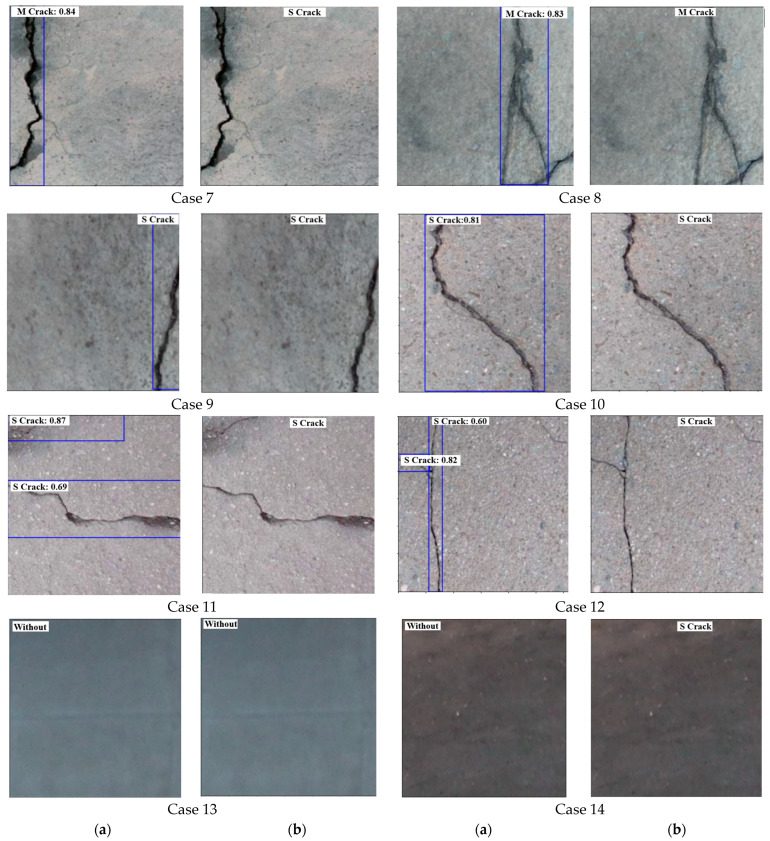
Comparison between the ViT individual model and the proposed Multi-stage YOLOV10-ViT model response: (**a**) Prediction based on YOLOV10-ViT model; (**b**) prediction based on ViT model.

Regarding the results of the multi-stage hybrid model (YOLOV10-VIT), the outcomes show the accuracy of the proposed methodology in classifying the predicted ROIs into ‘single crack’, ‘multi-branched crack’, or ‘without crack’ types. Figure 14 proves the efficiency and robustness of the proposed multi-stage YOLOV10-ViT model compared to the usage of the individual ViT model from two both points of view. First, the multi-stage YOLOV10-ViT model can detect crack ROIs within the image and then classify them to the appropriate class giving more precise information about the image and defining not only the class but also the possible ROIs that contain the crack region. The second point of view is the enhancement of the crack type classification by first detecting the possible defect parts and then classifying them instead of classifying the entire image. As introduced in Figure 14—Case 1, the test sample includes a sample with a ‘simple crack’ type. However, the ViT model misclassifies it as ‘Without crack’; on the other hand, the proposed multi-class model correctly classifies it as ‘Single crack’ and also produces the confidence level.

Cases 2 to 5 in Figure 14 also represent another situation in which the ViT model predicts samples as ‘Single crack’; while they are indeed ‘multi-branched crack’. However, the proposed multi-stage YOLOV10-ViT model has accurately classified these samples as a ‘multi-branched’ crack type. In case 6, the ViT model and the multi-stage model correctly classified the sample as a ‘multi-branched crack’ type. Cases 7 and 8 are both correctly classified by both models as ‘single crack’. Case 9 contains both types of cracks (‘single crack’ and ‘multi-branched crack’ which is impossible to predict by ViT. However, using the proposed multi-stage, the YOLOV10 first detected two bounding boxes, and then the ViT classified each one as a different class (i.e., one as a ‘single crack’ and the other one as a ‘multi-branched crack’). Although the proposed multi-model succeeded in most cases, but still some cases in which the multi-branched crack can be identified by the YOLOV10 model as two regions and classified as two ‘single cracks’ leading to a misclassification case. However, the individual ViT model fails to predict this sample. Cases 13 and 14 contain two ‘without crack’ samples which are correctly classified by the YOLOV10-ViT model, while the second one is incorrectly classified by the individual ViT model as ‘single crack’.

### 4.3. Individual and Multi-Stage Models Numerical Comparison

**Figure 15:**
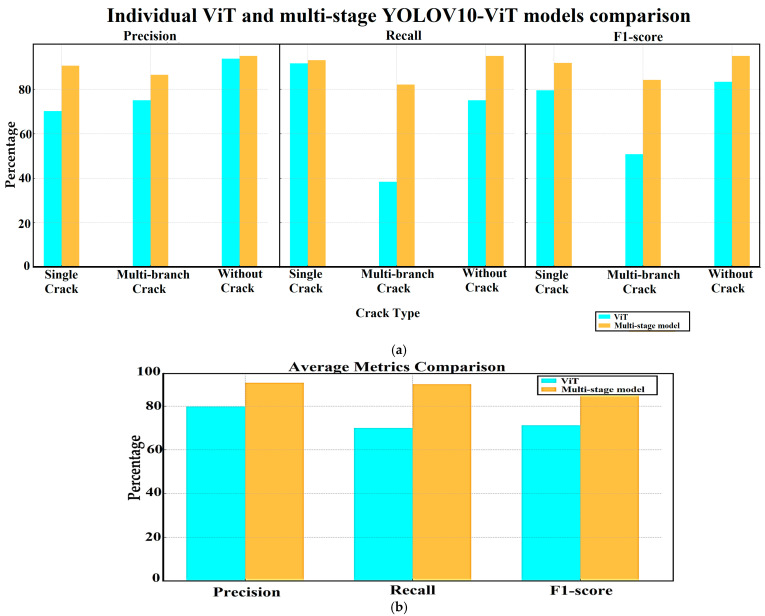
Comparison between the ViT individual model and the proposed Multi-stage YOLOV10-ViT model performance: (**a**) Precision, recall, and F1-score comparison; (**b**) average values comparison.

Although the individual model (ViT) has achieved a high performance on the utilized CICS dataset, it has acquired knowledge that is not sufficient to classify cracks in other various concrete crack datasets, which reduces its generality. In contrast, the proposed multi-stage model enhances the ability of the ViT model to correctly classify crack types even in the case of different image sources (various datasets) by focusing on specific regions in the image instead of classifying the entire image. Table 4 includes a comparison between both models in terms of using a concrete crack dataset test set which consists of 131 test images (distributed as follows: single crack: 72, multi-branch crack: 39, and without crack: 20 images), and the models are not trained or evaluated using this dataset in order to obtain more realistic results. Table 4 and Figure 15 prove that the multi-stage model outperforms the individual ViT model in terms of precision, recall, and F1-score metrics.

### 4.4. Ablation Study

In this section, some training hyperparameters of the ViT model are changed and the performance is evaluated again. All evaluation results of these training scenarios will be introduced and discussed.

#### 4.4.1. Changing the Input and Batch Size

**Figure 16:**
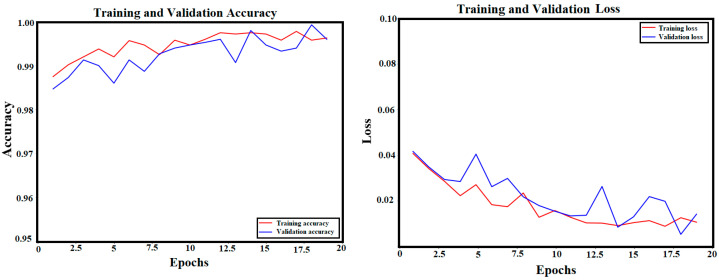
Training and validation curves of the new training parameters (patch = 16, input size = 224 × 224 × 3).

First, the input size of the training images is increased to 224 × 224 × 3 instead of 128 × 128 × 3 and the batch size is minimized to 16 instead of 32. Although these modifications improved the accuracy of the ViT model by 0.64% (from 99.29% to 99.93%), the training time has significantly increased from 126 s/epoch to 4.28 min/epoch. Figure 16 includes the training and validation results of the ViT model using 16 batch sizes and 224 × 224 × 3 input size.

#### 4.4.2. Fine Tuning the ViT Model

**Figure 17:**
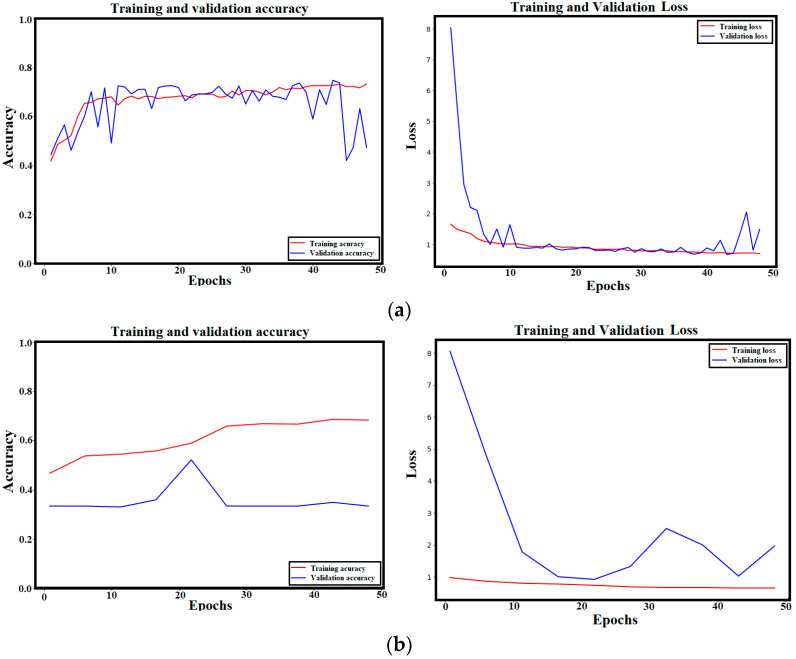
Training and validation curves resulted from training the entire ViT layers with eight multi-head attention layers and eight transformer layers (**a**) Lr = 0.0001, (**b**) Lr = 0.001.

Instead of using the pre-trained ViT model (freezing the feature extraction part’s weights), this experiment utilizes the ViT architecture and re-train the model entirely without loading weights. This experiment allows us to judge the ability of the ViT model to solve the crack-type classification problem without previous knowledge (older weights). However, Figure 17a shows that the re-training of the entire ViT model without loading its learnable parameters leads to a lower performance compared to the frozen-weights ViT model. The obtained validation accuracy is only 74.87% compared to 99.29% using the learned parameters of the ViT model (freezing layers). This issue happens when the utilized dataset is small compared to the dataset that the original ViT was trained by. Moreover, changing the learning rate (Lr) to 0.01 results in a worse performance (as shown in Figure 17b).

Furthermore, another trial is performed using a lower learning modified ViT architecture. In the modified architecture, only 8 transformer layers and 8 multi-head attention layers are utilized instead of 12. The outcomes indicated a lower performance as seen in Figure 17b. The validation accuracy of the modified ViT model with an Lr = 0.0001 is 74.87% compared to only 52.13% in the case of using Lr = 0.001 which makes the model unstable as shown in the training process (Figure 16).

#### 4.4.3. Changing the Learning Rate and Optimizer

Utilizing the main architecture of the ViT model with a learning rate value of 0.01 and Adam optimizer results in an overfitting case in which the validation accuracy is stuck at 33.33% without any improvement during the training process. Changing the Lr value to 0.001 results in 75.6% validation accuracy after 15 epochs (with a stopping condition). Utilizing a learning rate of 0.0001 solves the overfitting problem and improves the validation accuracy to 99.29%. Reducing the learning rate to 0.00001, the validation accuracy registers a maximum value of 99.9%. Further, two other different optimizers are utilized to compare their performance to the ‘Adam’ optimizer. The ‘RMSprop’ and ‘SGD’ optimizers are utilized with a fixed learning rate value of 0.0001.

**Figure 18:**
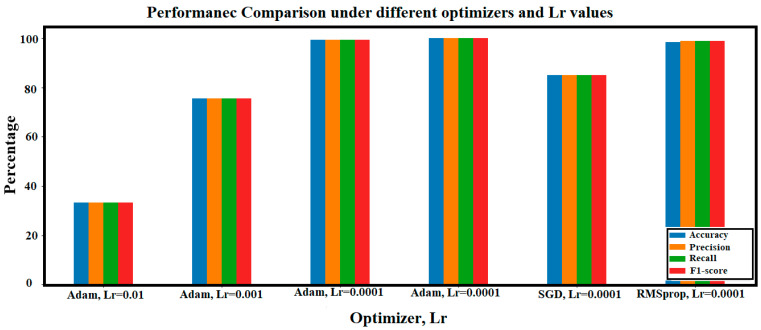
Accuracy, precision, recall, and 1-score comparison of training ViT model using different optimizers and learning rates.

Table 5 and Figure 18 illustrate the comparison between these optimizers and various learning rate values. Figure 18 shows that the best case corresponds to the utilization of the ‘Adam’ optimizer with a learning rate of 0.00001. This case has achieved 99.9% accuracy, precision, recall, and F1-score. However, the second-best case includes the utilization of the same optimizer but with a 0.0001 learning rate. This case also involves less computational time compared to the utilization of 0.00001 as a learning rate.

#### 4.4.4. Changing the Stop Condition

**Figure 19:**
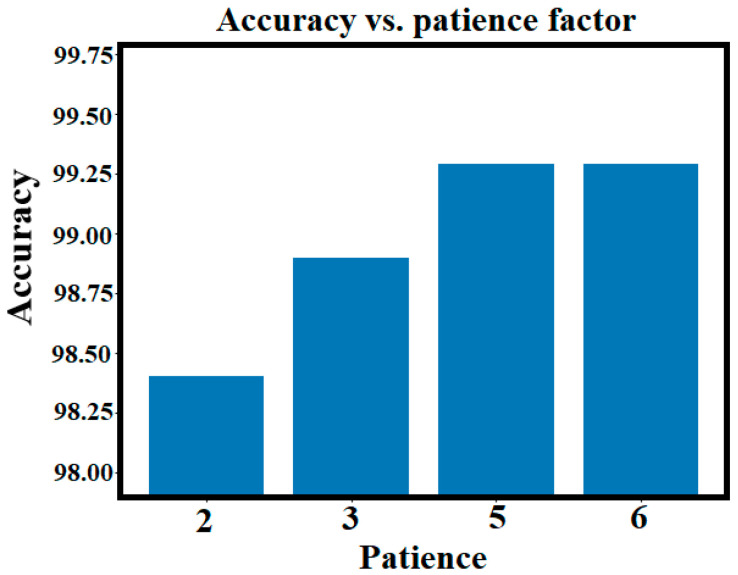
Accuracy comparison of the ViT-trained models under different patience factor values.

The value of the patience factor for early stopping of the training process was chosen based on three concepts: First, to avoid premature stopping which allows the model to stop at the best-performing state. Second, to prevent overfitting. Third, to limit unnecessary epochs where no performance gain is achieved. To ensure the robustness, we repeated the evaluation process with patience values (2, 3, 5, 6) and found that the best patience value that achieves the best performance is five. Higher values of the patience factor resulted in more training epochs without further enhancement. Figure 19 shows that the best case corresponds to the utilization of a patience value of five. The training process stopped at iterations 7, 14, 46, and 50 for the four cases of patience factor (2, 3, 5, and 6), respectively.

#### 4.4.5. Changing the Hyperparameters of the YOLO Model

**Figure 20:**
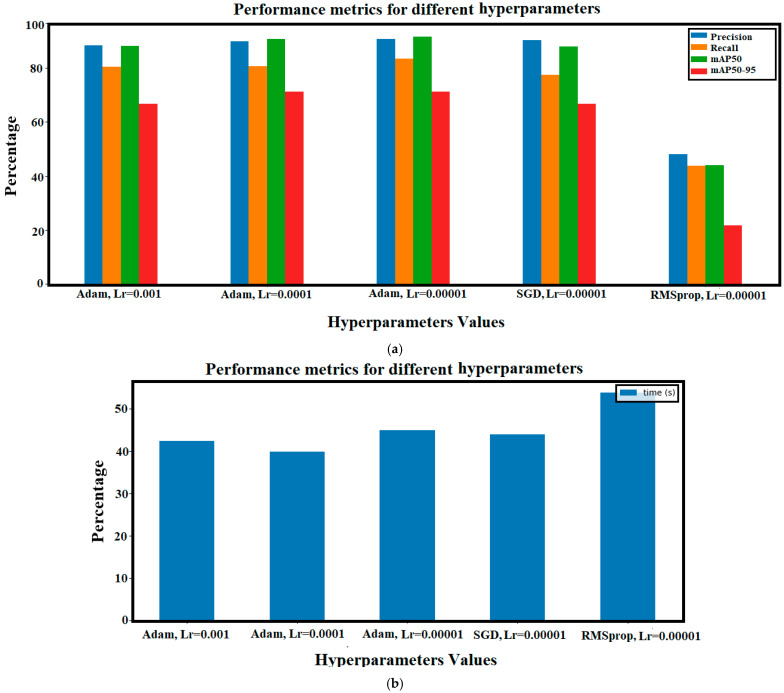
Performance comparison of YOLO model under different hyperparameter values: (**a**) evaluation metrics; (**b**) time.

In this part, the optimizer and the learning rate of the YOLO model are modified and as a result, five various experiments are applied. Figure 20 includes the outcomes of these experiments. The best choice of these hyperparameters is the case in which the ‘Adam’ optimizer and the learning rate value 0.00001 are chosen. The registered precision, recall, mAP50, and mAP50-95 values are 90.7%, 83.4%, 91.6%, and 71.3%, respectively. Figure 20b illustrates the consumed time required for training each of the various YOLO models. The best time corresponds with the utilization of the ‘Adam’ optimizer and 0.0001 as a learning rate value.

### 4.5. Limitations

**Figure 21:**
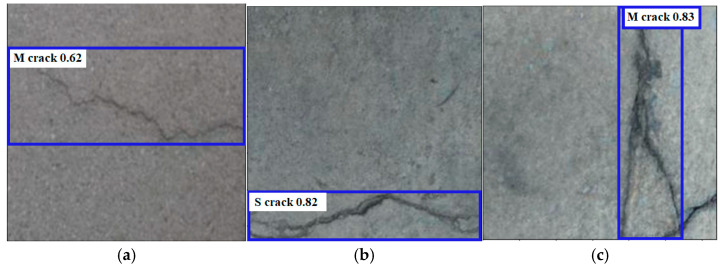
Some misclassified/miss detected samples: (**a**) Single crack case, (**b**) multi-branched crack case, and (**c**) multi-branched crack case with small undetected crack region.

Although the proposed multi-stage YOLOV10-ViT model outperformed the individual ViT model in all performance metrics, it still has some limitations. Figure 21 presents three cases of misclassification of the proposed YOLOV10-ViT model. The first case (a) represents a simple crack but the model misclassifies it as a multi-crack type. The second case (b) represents a multi-branched crack, but the model considered it as a single crack sample. However, the third case is a correct classification example, but the YOLOV10 model missed a small part of the crack although that does not affect its final correct decision. The main reason for the first two errors is due to the different sizes that will result from the YOLOV10 detection step which will be later resized to a squared shape to fit the input size of the ViT model. Future studies can focus on this limitation and try to use a partitioning approach that divides the detection result into correct squared shapes, trains different ViT models with different input sizes, and provides the YOLOV10 detection results to the ViT model with the closest input size.

### 4.6. Comparison with Previous Work

A comparison with the current state-of-the-art multi-stage concrete crack identification methodologies is presented in Table 6.

Table 6 proves that the proposed multi-stage system is novel compared to the previous multi-stage models which all focused on different applications or utilized multiple stages for only one mission (detection for example). Some studies utilized many algorithms for a specific mission and few studies targeted two steps (segmentation and classification for example). This study has targeted two steps to build a robust detection and classification model that does not classify the entire image but detects all possible crack regions inside the image and classifies them to the correct class.

## 5. Conclusions

The current research has comprehensively proposed and evaluated a multi-stage detection and classification deep learning-based model called the YOLOV10-ViT. The main aim of the developed model is to enhance the performance of concrete classification models by adding a pre-detection step that utilizes the YOLOV10 model to detect all possible crack-occurrence regions and then feed them into a trained ViT model to locally classify crack regions into either ‘single crack’, ‘multi-branched crack’, or even ‘without crack’ types. The ViT model has been trained using the CICS concrete crack dataset, while the YOLOV10 model has been trained using the “concrete crack detection dataset”. The multi-stage model has been evaluated using a test set of concrete crack images different from both datasets to correctly assess the ability of the proposed multi-stage model. Results have revealed that the ViT model achieved a test accuracy of 99.29%, while the YOLOV10 model registered a mAP50 score of 0.88139. Moreover, the proposed multi-stage model has successfully enhanced the performance of the individual ViT model by 10.9%, 19.99%, and 19.2% for precision, recall, and F1-score, respectively.

The proposed multi-stage YOLOV10-ViT model presents an efficient solution for a wide range of applications related to the construction industry, particularly after considering its boosted accuracy and performance. By integrating the developed model into construction systems, it is possible to significantly improve the safety and reliability of concrete structures by early detection of concrete cracks. The current trained model can be implemented in any automated construction operations in order to preserve the safety of the concrete materials and detect possible cracked parts of them. Furthermore, the model’s adaptability to various construction environments and structures and its capability to tackle various lighting conditions make it a robust and practical choice for in-practice tasks.

Although this study is the first one that develops a multi-stage model based on both the YOLOV10 and ViT models to enhance the ability of concrete class classification, it still has some limitations that can be addressed in the next studies. Firstly, the proposed methodology has given some false positives and false negatives due to the problem of different resizing options of YOLOV10 and ViT models. The second issue is the need to evaluate the proposed study on other crack-based datasets like bridge, wood, and other materials cracks. Next studies can also obtain the benefit of semantic segmentation models like U-Net to develop a multi-stage model based on a pre-segmentation step to further improve the classification performance. Since the current study is limited to the utilization of only ViT and YOLOV10 models, future work can also concentrate on the utilization of other deep learning frameworks.

In the case of some complex crack patterns, there can be a misidentification of multi-branch cracks into multiple single cracks. This can be solved by developing some algorithms for merging boxes that are spatially close to each other which can be misclassified as single cracks. Moreover, clustering algorithms can be applied to cluster the detected cracks based on their location and visual similarities which can help to identify potential multi-branch cracks.

## Figures and Tables

**Table 1:** The YOLOV10 model training parameters.

Parameter	Value
Image size	640 × 640
Epochs	50
Batch Size	32
Learning Rate	0.001
Optimizer	Adam
Number of classes	2 (Crack region and Background)

**Table 2:** The ViT model training parameters.

Parameter	Value
Image size	128 × 128 × 3
Test size	20%
Batch size	256
Activation (Decision layer)	Softmax
Pretrained ViT	True
Number of classes	3
Optimizer	Adam
Loss function	Categorical Cross entropy
Learning rate	0.0001
Early stop condition	Yes
Early stop condition patience	5
Epochs	50

**Table 3:** Performance metrics of the trained ViT model.

Class	Precision (%)	Recall (%)	F1-Score (%)
Multi-branched crack	99.4	99.88	99.64
Simple crack	99.24	98.73	98.98
Without crack	99.23	99.23	99.23
Macro Average	99.29	99.28	99.29
Weighted Average	99.29	99.29	99.29

**Table 4:** Correct and misclassification samples registered by both individual ViT and multi-stage YOLOV10-ViT models.

Samples/Models		ViT	Multi-Stage YOLOV10-ViT	Metrics (ViT)	Metrics (Multi-Stage)
‘single’ crack test samples	Correct classification	66	67	**P:** 66/(66 + 24 + 4) = **70.21%** **R:** 66/(66 + 6) = **91.66%** **F: 79.51%**	**P:** 67/(67 + 6+1) = **90.54%** **R:** 67/(67 + 5) = **93.05%** **F: 91.77%**
	Misclassified as Multi-branch crack	5	5		
	Misclassified as without crack	1	-		
	Total	72	72		
‘multi-branch’ crack samples	Correct classification	15	32	**P:** 15/(15 + 5) = **75%** **R:** 15/(15 + 24) = **38.46%** **F: 50.84%**	**P:** 32/(32 + 5) = **86.48%** **R:** 32/(32 + 7) = **82.05%** **F: 84.2%**
	Misclassified as Single-crack	24	6		
	Misclassified as without crack	-	1		
	Total	39	39		
‘Without crack’ samples	Correct classification	15	19	**P:** 15/(15 + 1) = **93.75%** **R:** 15/(15 + 5) = **75%** **F: 83.33%**	**P:** 19/(19 + 1) = **95%** **R:** 19/(19 + 1) = **95%** **F: 95%**
	Misclassified as Single-crack	5	1		
	Misclassified as Multi-branch crack	-	-		
	Total	20	20		
Average Metrics				**P:** 79.77% **R:** 70.04% **F:** 71.22%	**P:** 90.67% **R:** 90.03% **F:** 90.34%

P: precision, R: Recall, and F: F1-score.

**Table 5:** Comparison of different learning rates and optimizers trials (bolded values are for the best score).

Class	Accuracy (%)	Precision (%)	Recall (%)	F1-Score (%)
’Adam’, Lr = 0.01	33.33	33.33	33.33	33.33
’Adam’, Lr = 0.001	75.6	75.5	75.6	75.54
’Adam’, Lr = 0.0001	99.29	99.23	99.23	99.23
’Adam’, Lr = 0.00001	**99.9**	**99.9**	**99.9**	**99.9**
’SGD’, Lr = 0.0001	85.07	85.07	85.07	85.07
’ RMSprop’, Lr = 0.0001	98.4	98.93	98.93	98.93

**Table 6:** Comparison with previous multi-stage concrete crack identification methodologies.

Researcher	Multi-Stage Technology	Methodology	Dataset	Results	Limitations
Yadav et al. [19]	Multi-feature extraction parts	LBP, iterative clustering, 3ScaleNetwork	Historical_Building_Crack_2019 3886 (757 crack images)	Precision: 98.9%, Recall: 99.18%, Accuracy: 99.69%	Unstable performance due to unbalanced dataset
Chen et al. [20]	Multi-resolution segmentation methodology	multi-resolution semantic segmentation	2000 images of bridge, dam, and spillway materials	Precision: 94.51%, Recall: 86.39%	Small dataset, High false negative ratio
Wang et al. [29]	Integrating ViT as backbone for YOLO	YOLOV8 with ViT backbone	1994 asphalt pavement images	Precision: 87.2%	Too many false positive errors
Nguyen et al. [30]	Crack detection and segmentation	CNN, U-Net	DeepCrack: 537 images The CrackIT: 84 pavement surface images	F1-score: 91%	Small dataset size with some FP and FN errors
Dai et al. [32]	Damage detection	machine learning and piezoelectric singular feature analysis	A signal-based dataset of 1344 signals	Accuracy:92%, Precision: 95.4%, Recall: 92.9%	Small dataset size, stages considered only the detection
Chen et al. [33]	Crack segmentation and quantification	U-Net and multi-task DeepLabV3+	DeepCrack dataset	Improved the concrete crack quantification by above 2%	High computational time compared to mall improvement
Current study	Detection and classification	YOLOV10 and ViT	CICS: 12,000 images, Crack in various Materials from Historic Buildings: 1116 images	ViT: accuracy: 99.93% YOLOV10: Precision:90.7%, Recall: 83.4%, mAP50: 91.6% Multi-stage: precision: 90.67%, Recall: 90.03%, F1: 90.34%	Some errors happen due to different sizes of the detected crack regions

## Data Availability

These data were derived from the following resources available in the public domain: [https://data.mendeley.com/datasets/9brnm3c39k/1] (accessed on 1 August 2024).
